# Data on degradome sequencing and analysis from mock-inoculated and *Fusarium oxysporum* treated leaves samples in *Persicaria minor*

**DOI:** 10.1016/j.dib.2018.08.034

**Published:** 2018-08-20

**Authors:** Abdul Fatah A. Samad, Muhammad Sajad, Jaeyres Jani, Abdul Munir Abdul Murad, Ismanizan Ismail

**Affiliations:** a School of Biosciences and Biotechnology, Faculty of Science and Technology, Universiti Kebangsaan Malaysia, 43600 UKM Bangi, Selangor, Malaysia; b Institute of Systems Biology, Universiti Kebangsaan Malaysia, 43600 UKM Bangi, Selangor, Malaysia; c Borneo Medical and Healthy Research Center Faculty of Medical and Healthy Science, Universiti Malaysia Sabah, Malaysia; d Department of Plant Breeding and Genetics, University College of Agriculture & Environmental Sciences, The Islamia University of Bahawalpur, Punjab, Pakistan

## Abstract

Degradome sequencing referred as parallel analysis of RNA ends (PARE) by modifying 5′-rapid amplification of cDNA ends (RACE) with deep sequencing method. Deep sequencing of 5’ products allow the determination of cleavage sites through the mapping of degradome fragments against small RNAs (miRNA or siRNA) on a large scale. Here, we carried out degradome sequencing in medicinal plant, *Persicaria minor*, to identify cleavage sites in small RNA libraries in control (mock-inoculated) and *Fusarium oxysporum* treated plants. The degradome library consisted of both control and treated samples which were pooled together during library preparation and named as D4. The D4 dataset have been deposited at GenBank under accession number SRX3921398, https://www.ncbi.nlm.nih.gov/sra/SRX3921398.

**Specifications Table**

**Table:** 

Subject area	Biology
More specific subject area	Biotechnology
Type of data	Table and public repository
How the data is acquired	Ilumina sequencing (HiSeq. 2500)
Data format	Raw and analyzed
Experimental factors	Controlled growth chamber and *Fusarium oxysporum* treated
Experimental features	Degradome sequencing
Data source location	Selangor, Malaysia (3° 16′14.63″ N, 101° 41′ 11.32″ E)
Data accessibility	The raw data can be accessed at public repository site (https://www.ncbi.nlm.nih.gov/sra/SRX3921398). The analyzed data are available in this article.
Related research article	Samad, A.F.A., Nazaruddin, N., Sajad, M., Jani, J., Murad, A.M.A., Zainal, Z. and Ismail, I. (2017). Small RNA sequencing for secondary metabolite analysis in *Persicaria minor*. Genomics Data 13, 3–4 [1]

**Value of data**

• This data is the first degradome library in *Persicaria minor*, where the genome information still unavailable.
• Degradome technique is the latest technology for determination of cleavage site of small RNA.
• Identification of miRNA-target interaction is vital in understanding the biology of the miRNA regulatory mechanism.

## 1 Data

The small RNA data in the article [1], combined with degradome data in this present article, will provide a comprehensive insight into the cleavage site assessment of small RNA. In total, about 17,532,759 reads was generated (Table 1). Filtering the low read sequences and adaptor removal produced 15,493,710 clean reads. Mapping of the clean reads to reference gene retrieved 3,074,840 (19.85%) sequence tags. Sequence tag referred to degradome fragments that were used for the determination of small RNA cleavage sites. In addition, about 2,039,049 (15.48%) reads were discarded during the filtering process due to low quality reads.

**Table 1:** Degradome statistic for D4 library.

	Total number of reads	Percentage (%)
Raw reads	17,532,759	100.00
Discarded reads	2,039,049	15.48
Clean reads	15,493,710	100.00
Sequence tag	3,074,840	19.85

## 2 Experimental design, materials and methods

### 2.1 Plant materials and treatment

*P. minor* explants were cultured and grown as mentioned in the previous work [1]. A set of *P. minor* plants that consist of mock-inoculated and *Fusarium*-treated were prepared. The fungus treatment was carried out as mentioned in [2].

### 2.2 RNA extraction, quality control and library preparation

Plant RNA Reagent (Invitrogen, USA) were used to isolate total RNA from both samples according to recommended protocol. Then, both RNA samples were pooled together in one tube and named as D4. Quality control and library preparation for D4 was carried out according to previous report [1], [3].

### 2.3 Raw reads pre-analysis and mapping

Trimming of adapter index sequences were carried out using Skewer software (https://sourceforge.net/projects/skewer) [4]. Additionally, low quality reads were removed to produce clean reads. Mapping of degradome fragments against reference gene were carried out using psRobot software [5], [6]. Overall statistical result for D4 was summarized in Table 1.
