# Supplementary material for: Data on degradome sequencing and analysis from mock-inoculated and Fusarium oxysporum treated leaves samples in Persicaria minor
Source: Data Brief. 2018 Aug 20;20:555–7. doi: 10.1016/j.dib.2018.08.034 (PMC6126405; doi:10.1016/j.dib.2018.08.034)
Supplement: Supplementary file 1 — Supplementary material [file mmc1.docx]

Conflict Of Interst Form

The authors declare that the research was conducted without any commercial or financial benefit that could be taken as a potential conflict of interest.
